# A New Look at Teenage Pregnancy in Brazil

**DOI:** 10.5402/2011/975234

**Published:** 2011-09-05

**Authors:** Maria Luiza Heilborn, Cristiane S. Cabral

**Affiliations:** Institute of Social Medicine, State University of Rio de Janeiro (IMS/UERJ), Rio de Janeiro, Brazil

## Abstract

This paper brings a synthesis of some of the main results provided by GRAVAD survey (Teenage pregnancy: multicentric study about youth, sexuality and reproduction in Brazil). GRAVAD is a study about sexual and reproductive behavior among Brazilian youth that interviewed 4,634 individuals in a population survey with a random sample. Women and men between 18 and 24 years old were interviewed in three capitals—Porto Alegre, Rio de Janeiro and Salvador. “Teen pregnancy” is not the consequence of promiscuous sexual activities, as popular beliefs currently state. It is often ignored that amidst the poorer social segments parenthood is seen as a sign of social status, given the lack of professional perspectives. Among the middle class, other sociocultural horizons give parenthood the status of an experience to be lived in later moments of live, when one's professional and financial lives have been consolidated.

## 1. Introduction

Since the second half of the 20th century, when the process of separation between sex and procreation began, a profound transformation of customs has affected men and women's behavior as the preservation of virginity until marriage loses its moral significance. In this context of cultural changes, the phenomenon of “teen pregnancy” has emerged as a disturbing element in ideal youth development. This paper summarizes the main results provided by GRAVAD survey (teenage pregnancy: multicentric study about youth, sexuality, and reproduction in Brazil) [1]. It allows both to problematize the moralistic tone of common sense belief that tends to treat the phenomenon as homogeneous and contemporary and to designate a new outline for the comprehension of reproductive events in juvenile biographies.

In order to understand this change in mentality, it is important to focus the social processes that contributed to the construction of “teen pregnancy” as a “problem” [1–3]. Surrounding the expression “teen pregnancy” is the assumption about a certain age group that, for a long time, was seen as ideal for a woman to have children [4, 5]. However, current debate treats it as an “early” event in youth trajectory and as an exclusive characteristic of poorer segments of the population [6–8]. 

Demographic transition has been identified as one of the central elements in the intriguing panorama of “early reproduction” [2, 9, 10]. According to the 1970 census, the total fertility rate (TFT) in Brazil was 5.76 children per woman, arriving at 1.81 in 2010. Traditionally, the higher rates of fertility (age-specific fertility rate) were seen in women from 25 to 29 years or from 30 to 34 years, constituting what is commonly referred to as a later pattern of fertility. Since the 1980s, there has been an observed growth in fertility rates among women between 15 and 19 years old, a datum that becomes notable when compared to the simultaneous fall in the rates of other age groups [2, 9, 10]. This dislocation is responsible for a typically young pattern in the fertility structure by age groups in Brazil, a pattern that is different from what it is observed in developed countries [9].

Although the increase in the fertility rate of women between 15 and 19 years old is not statistically significant and has in fact been the effect of profound reductions in the participation of the oldest women in the total fertility rate [10], the data mentioned above have been used to set the scene for a pregnancy epidemic in adolescence, above all in the poorer segments of the population [11]. The public debate in relation to the phenomenon frequently and erroneously associates teen pregnancy with questions of poverty and urban violence in the country [3, 11]. 

Within the scenario of changes in society, there have also been transformations in the social conceptions of age and gender and these transformations redefine the social expectations placed on young men and women today [12]. Ages are not natural and their conceptions vary socially and historically. Nowadays, it is expected that adolescents and youth dedicate themselves to education in order to achieve insertion into professional life—these two elements are important demarcations for the passage to adult life [12]. According to this model, leaving parental home and beginning conjugal life would conclude this transition to adulthood [12]. 

This ideal conception of passage to adult life ignores that the availability of social opportunities is not offered in equal conditions for youth of different social classes. Furthermore, there is no homogeneity in how to experience adolescence and youth. According to the current rationale of the ideal transition to adulthood, an episode of pregnancy in this life period is interpreted as disturbing youth development, as it accelerates or even suppresses some phases taken as “natural” in life course [1, 4–6]. Moreover, this rationale obliterates the fact that there are gender and class differences that interfere in the process of sexual initiation [13].

## 2. Material and Methods

GRAVAD is a study about sexual and reproductive behavior among Brazilian youth. Young people were interviewed in three major Brazilian State capitals: Porto Alegre (Rio Grande do Sul), Rio de Janeiro, and Salvador (Bahia). These cities are located in regions with very different social and cultural characteristics (the Northeast, Southeast, and South, resp.). 

The research approach in this study links two methodological strategies: semistructured interviews (*n* = 123), carried in 1999-2000, and a household survey (taken place in 2002) with a three-stage stratified probabilistic sample of men and women from 18 to 24 years of age. 

Face-to-face interviews were held with a questionnaire based on the results of the qualitative stage. The instrument has the same list of questions for both sexes. Questions were worded according to the interviewee's sex. The questionnaire prioritized certain events and situations in the individual's affective and sexual history, such as first sexual relation, first and most recent pregnancy, first and most recent child, first abortion (spontaneous and induced), and current partner.

## 3. Results and Discussion

### 3.1. Changes in Social Customs

The changes in sexual customs taking place in Brazil, as well as in western countries, have accepted preconjugal feminine sexuality. The first sexual experiences, originally allowed to young men but only with specific partners, such as prostitutes or “women who were not worth of marrying with,” began to happen with girlfriends. Paradoxically, despite an environment of transformations in which sex gains a status among youth as an acceptable behavior, conversations about sexuality continue to be taboo in the family; contraception is not openly discussed in school, and sexual education is a highly controversial theme in Brazilian society. Teen and youth sexual relations have been modified, but these changes were not sufficient to alter the ways in which contraception can be discussed. Women are still considered to be the sole responsible for pregnancy, while men continue being absolved or omitted from their participation in the reproductive event [14].

In Brazil, male initiation continues to be more precocious than women's by at least two years (16.2 against 17.9 years)—this is a pattern commonly found in Latin America [7]. Gravad data show that the behavior of young men tends to be more uniform (see Table 1): for example, the median age for their first sexual experience does not vary according to region of residence, social group, or skin color/race. However, there is a delay of this initial experience in the context of prolonging individual education [13].

Women present a greater diversity of conduct, according to their family origins and their biographic characteristics. Those from poorer groups, who also share other characteristics such as a lower level of education and socialization with significant involvement in domestic work, have an earlier sexual initiation [13, 15]. Contrary to the prevailing stereotypes in Brazilian society with respect to ethnic groups, there was no observed greater precocity in sexual initiation among black women [13].

Some characteristics of the partner at first sexual relationship are presented in Table 2. The results provide by GRAVAD show that there is a recurrent age difference between partners at the first sexual experience: women have older and more experienced partners [13]. They have their first experience with the *namorado* (steady boyfriend) (86%) or the husband (4%). Barely 45% of men have their first experience with the girlfriend; half of men do have their first experience with an eventual partner, and 5% with prostitutes. The use of contraceptive methods also varies according to the age of the first relation; it is higher among youth who begin their sexual experiences later. The degree of use also varies according to the level of individual instruction, being higher among those with higher levels of education [13]. Although equivalent proportions of men and women (70%) say they have used some form of contraception for their first sexual intercourse, we observed a decline in regular use of contraceptives afterwards, which is associated with the type of relationship between partners [13, 16].

It is common to associate the occurrences of an unplanned pregnancy with the nonuse of contraceptive methods, which is seen as a consequence of lack of knowledge or difficulty in accessing contraceptive resources on the part of young people. Notwithstanding, this panorama in which young women have a sexual initiation with an older or more experienced partner—the* namorado*—exercises a strong influence on the decisions regarding contraceptive protection [17, 18]. Being informed about the existence of and the way to use contraceptive methods is not sufficient to guarantee their adequate use. It is important to take into account the context of gender relationships, which influences the contraceptive practices. The hierarchy of gender influences the decision to use condoms. And it is expected that young women take on all of the responsibility with respect to contraception, with a low level of participation from their male partners. 

### 3.2. Heterogeneity in Reproductive Experience during “Adolescence”

Considering adolescence as comprising the ages between 10 and 19 [19], GRAVAD data show that a first experience with “teen pregnancy” was declared by 21% of men and 29% of women (proportion calculated with those that were 20 years old or above) [15, 20]. When working with the cutoff point of 18 years of age—which constitutes the starting age of Brazilian legal adulthood—the observed proportion is lower: the event is reported by 8% of men and 16% of women. Below the age of 15, the percentages are much smaller: 0.6% for men and 1.6% for women [15, 20]. 

The features of these pregnancies indicate that they occurred in steady relationships; there is a small percentage of those interviewed saying that their first episode of pregnancy before age 20 was with an eventual partner. In this first pregnancy, most young women's partner was the same person with whom they had their first intercourse (56%), which contrasts with 21% of the young men in the same condition. Greater proportions of pregnancy before age 20 were observed among those youth with lower education levels and those who were not working at the time of the interview. As for the young men involved in pregnancy episodes with their partners, the majority of them are already working, albeit informally, when the event took place [15, 20, 21]. 

The level of young women's engagement in domestic work constitutes an important factor in their socialization. There is a clear association between teen pregnancy and the fact that young women are the main ones to perform the domestic work in their families of origin [15, 20], which indicates that family and gender socialization delineate the horizon of biographic trajectories [17, 22, 23]. Intense involvement with the domestic sphere promotes a determined vision of the world, in which being a mother is a central element of feminine identity—a well-documented characteristic in the anthropological Brazilian literature about families [22–24]. It was observed, moreover, that teen pregnancy was more referred by youth whose mothers and siblings had children before age 20. For this reason, this seems to be an accepted and reproduced behavior in their environment, which does not deviate from the representation that prevails in the family of origin. 

Despite family socialization, individual education is certainly a relevant factor that favors the postponing of entry into sexual life with a partner [13]. It also reduces the chances of a so-called early pregnancy [13, 21, 25]. In every social stratum that was studied, the reference to an episode of pregnancy before age 20 diminishes in the same proportion as the education of the young man or woman increases [20, 21, 25]. Education widens personal and professional horizons, even in the face of a strong social disadvantage, as well as relocates the goals of motherhood and fatherhood.

The hidden side of the debate regarding the phenomenon of teen pregnancy is the debate about abortion [26, 27]. In Brazil, abortion is prohibited except for two specific circumstances: rape and life risk to the mother. As can be seen in Table 3, the survey permitted us to ask questions regarding the interruption of pregnancy in youth: more women than men affirmed having carried their first pregnancy to term, when the pregnancy occurred before age 20, even though the decision had involved conflicts and negotiations with their partners and families [20, 26]. The experience of having had a child while young is strikingly distinct for young men and women: among those who have had at least one teen pregnancy, 50% of men and only 16% of women did not have any children at the time of the interview [20, 26]. The fact that many studies erroneously take pregnancy and maternity as synonyms ends up obliterating the cases of abortion and fatherhood among youth [1].

### 3.3. Youth Parenthood: The Impacts according to Gender and Class

Early reproduction is seen as a problem for the life of young people because of the social expectations driven to youth, such as the increase of their education and the postponement of reproduction [1, 4]. Nevertheless, youth with children present a set of specific sociobiographic characteristics that suggests a rapid passage to adult life, wherein the reproductive episode accelerates the process or even represents its conclusion [24, 28]. For this reason, they contrast with middle/upper class youth, among whom the extension of the transition to adulthood is observed, whether through prolonged studies and/or their permanence in their parents' home [29]. It must be added that among youth in middle and higher social segments, the episodes of teen pregnancy tend to end in abortion [26]. 

The impacts of the birth of a child during youth on the trajectories of young women and young men are very different, and they are very much affected by class condition [24, 28, 29]. The process of decision making with respect to pregnancy goes beyond the limits of the couple [19, 30]. The families of origin play a fundamental role in decisions related to maintaining the pregnancy or not, establishing a conjugal union, and providing the material and financial resources that will sustain the child, and so on. Young people generally turn to their parents for the support of the arrival of the future grandchild. As can be seen in Table 4, the family reaction is generally positive when told the news [24]. In the metropolitan areas covered by the survey, the situation where a young woman is expelled from the parental home because of a pregnancy is rare, which means a relevant change regarding sexual morality and family/intergenerational relationships [22, 29, 30]. The families of origin represent an important network of support for the new couple, whether that is through housing them in their residences, contributing with goods or taking care of the child [29, 30]. 

Cohabitation with the partner as a result of the reproductive episode is frequent in the trajectories of lower-income youth [30]. For the middle class, conjugal alliance is not valorized in the context of juvenile reproduction [29]. When the gestation is carried to term, families mobilize in support of maintaining the project of the youths' education (with nannies or daycares), without this implying the acceleration or entry of the youth into the labor market. The children of these young people find themselves mainly in two situations: they either live with both parents or with the woman and her family. Domestic arrangements with the mother as a single parent are rare in this age group. In the cases of dissolution of the couple, the young women and the children return to their families of origin [15, 20, 24, 29, 30]. 

It is important to highlight the repercussions of motherhood or fatherhood on these youth's schooling and job trajectories: Table 4 shows that more than 60% of young men were already in the labor market before the birth of their first child; only 14% did not have any type of work activity and remained jobless. Besides working, half were already out of school and 15% declared that they had abandoned their studies. The majority of the women did not participate in any labor activity for remuneration (65%), nor were they at school (40%), and 18% quit school with the birth of their child [21, 24]. 

Thus, it is important to note the existence of different juvenile trajectories, in which schooling is short and with frequent grade repetitions for many [21]. The reproductive event does not interrupt careers: for some, the social/family structure permits the continuity of education and job training; for others, this path has already been interrupted and the reproductive event is the closure of a transition towards adult life [15, 21, 24, 28]. 

The impacts of having children on the sphere of juvenile sociability constitute another relevant dimension. The impact is undoubtedly greater for young women: 72% of women declared having reduced the time spent with friends in the first year after the birth of the child, which reflects gender differences in the division of childcare. At this point, it is important to underscore that motherhood strengthens a “feminine innerness” linked to the domestic world: complaints of “loneliness” and “isolation” are common, especially among poorer young women [24]. Qualitative data indicate that, in comparison to their male cohort, middle class young women become more confined to the house; that is, they experience more restrictions in going out after the birth of a child. Second, a cross-class comparison shows that middle class women do go out more, receive more support for maintaining social relations, and do not experience reclusion as radical as that for young women in the lower socioeconomic segment. The latter are the ones who undertake duties with husband, child, house, domestic work, and so forth, and in whose lives gender asymmetries are added to social inequalities [29, 30].

## 4. Final Remarks

Adolescence is a period of life in which peers gain greater importance. This is part of the process through which youth simultaneously construct their autonomy in relation to their families and search for their singularities. The contemporary notion of “teen pregnancy” very often ignores this strong characteristic of juvenile behavior [3–5, 31]. Views disseminated about youth mostly refer to a negative evolution of customs and highlight a precocious and undesirable atmosphere of eroticization (usually attributed to the media) [32], as well as the irresponsibility of youth, their ignorance, the parents' lack of authority, and the absence of dialogue between the generations. 

From the point of view of common sense beliefs, teenage pregnancy is presented as a social problem in Brazil. On the one hand, there is a (false) perception of a demographic explosion among the poorer segments of society. However, Brazilian census data show a significant decrease in the fertility rates for the last 30 years. On the other hand, sexual experience before and outside of marriage ceased to be a privilege of young men. Nevertheless, the importance of gender hierarchy in presiding decisions about contraception has been little modified and the network of people and institutions supposed to provide sexual guidance has not increased [33]. Actually, sexual education continues to be a difficult topic to broach at home and at school. The option for avoiding the debate about contraception means to ignore the fact that sexual relations among youth and adolescents have changed [13]. 

The magnitude of social mobilization with respect to this so-called problem is intimately connected with changes in the social conceptions of age and gender. As already mentioned, these changes have produced social expectations linked to an adequate youth trajectory, which is translated in terms of greater levels of education and postponed reproduction [1, 12].

Another issue elucidated by GRAVAD survey is that “teen pregnancy” is not the consequence of promiscuous sexual activities, as common sense beliefs currently state. It is often ignored that amidst the poorer social segments motherhood is seen as a sign of social status, given the lack of academic or professional perspectives [24]. Differently, other sociocultural horizons among middle class youth give motherhood the status of an experience to be lived in later moments of live, when one's professional and financial lives have been consolidated [24, 29].

The phenomenon in question is not equally present in all social strata. It is concentrated among women with lower education levels who come from families with lower cultural and financial capital [15, 20, 25]. Becoming a mother constitutes a social horizon aspired to by many of these young women. Contrary to common sense beliefs that teen pregnancy is a result of poverty, early pregnancy is also present among youth in higher social strata. This means that it also occurs among those with access to information and contraceptive methods, with access to safe—albeit clandestine—abortion [26, 27] and with real possibilities of fulfilling life projects in which parenting has the status of something to be experienced at a later moment of life.

## Figures and Tables

**Table 1:** Median age at first intercourse and selected biographical characteristics, by sex.

Selected biographical characteristics	Median age at first sexual intercourse	
	Women	Men
City		
Porto Alegre (RS)	17,2	16,2
Rio de Janeiro (RJ)	17,8	16,1
Salvador (BA)	18,4	16,4
Total	17,9	16,2

Religion in which the person was raised		
Catholic	18,0	16,2
Protestant	18,1	16,9
Pentecostal	18,4	16,3
Spiritism	18,2	16,1
Afro-religions (*umbanda, candomblé, batuque*)	16,8	16,2
Other	18,8	15,3
No religion	16,3	16,4

Schooling		
Elementary incomplete	16,3	15,8
Elementary complete	17,5	15,9
Secondary	18,6	16,4
College	18,6	16,7

Race/skin color		
White	17,8	16,4
Black	17,8	15,9
Brown/mixed (*parda*)	18,1	16,1
Other	17,9	16,0

Source: Gravad, 2002.

Population: youth from 18 to 24 years old, Porto Alegre (RS), Rio de Janeiro (RJ), Salvador (BA).

**Table 2:** Characteristics of the partner at the first sexual relationship, by sex.

Characteristics of the partner at the first sexual relation	Sex (%)	
	Women	Men
Type of relationship		
*Namorado *(steady boyfriend/girlfriend)	86	45
Husband/wife/living together	4	1
Eventual partner	9	48
Prostitute	—	5

Was it also the partner's first sexual relation?		
Yes	14	37
No	83	57
Do not know	3	6

Age difference between the interviewee and the partner		
The partner is younger	2	10
Same age (±1 year)	24	52
The partner is older (2–4 years)	36	24
The partner is much older (5 years and more)	38	15

The partner was in school		
Yes	50	76
No	50	24

Source: Gravad, 2002.

Population: youth from 18 to 24 years old, Porto Alegre (RS), Rio de Janeiro (RJ), Salvador (BA).

**Table 3:** Distribution of youth from 18 to 24 years old (excluding virgins), according to the type of reproductive event in the sexual trajectory (pregnancy or child birth), by sex and city.

Type of reproductive event	Porto Alegre		Rio de Janeiro		Salvador		Total		*P* value
	*n*	%	*n*	%	*n*	%	*n*	%	
Women	631		651		718		2000		
Pregnancy		34,9		41,2		53,3		43,8	0,0040
Child birth		28,8		31,9		43,4		34,8	0,0079

Men	695		675		669		2039		
Pregnancy		26,3		31,1		35,5		31,7	0,2401
Child birth		16,8		16,7		16,0		16,5	0,9670

Source: Gravad, 2002.

Population: youth from 18 to 24 years old, Porto Alegre (RS), Rio de Janeiro (RJ), Salvador (BA).

**Table 4:** Distribution of youth from 18 to 24 years old who have child/children, according to the characteristics of the first pregnancy carried to term and the outcomes through the first year after the birth of the child, by sex.

Selected characteristics	Women		Men		Total		*P*-value
	*n*	%	*n*	%	*n*	%	
Main reaction of the family with the news of the pregnancy	625		240		865		0,0072
The family was happy		45,1		43,1		44,5	
The family offered help in taking care of the child		21,8		34,8		25,8	
The family suggested an abortion		9,4		6,2		8,4	
The family demanded marriage/conjugal union		8,6		3,4		6,9	
The family expelled you from home		3,9		0,3		2,7	
The family did not know about the pregnancy		2,5		4,9		3,3	
Other reactions		8,8		7,5		8,4	

Schooling in the first year after delivery	617		237		854		0,0016
Not in school, and continued out of school		47,1		53,3		49,0	
Continued in school		14,8		26,0		18,3	
Quit school for a period		22,7		12,2		19,5	
Definitively quit school		15,4		8,5		13,3	

Work in the first year after delivery	620		235		855		0,0000
Not working and continued as such		59,3		12,0		44,7	
Started to work		14,0		18,4		15,3	
Quit working		8,2		4,2		6,9	
Already working		18,5		65,4		33,1	

With whom the first child lives	620		237		857		0,0000
With both parents		50,5		48,9		50,0	
With you and/or your family		38,9		5,1		28,4	
With the partner and/or her(his) family		1,9		41,2		14,1	
Other arrangements		8,7		4,7		7,4	

Source: Gravad, 2002.

Population: youth from 18 to 24 years old, Porto Alegre (RS), Rio de Janeiro (RJ), Salvador (BA).
